# Mass spectrometry in cerebrospinal fluid uncovers association of glycolysis biomarkers with Alzheimer’s disease in a large clinical sample

**DOI:** 10.1038/s41598-023-49440-3

**Published:** 2023-12-16

**Authors:** Matthijs B. de Geus, Shannon N. Leslie, TuKiet Lam, Weiwei Wang, Florence Roux-Dalvai, Arnaud Droit, Pia Kivisakk, Angus C. Nairn, Steven E. Arnold, Becky C. Carlyle

**Affiliations:** 1 Department of Neurology, Massachusetts General Hospital, Charlestown, MA USA; 2 Leiden University Medical Center, Leiden, The Netherlands; 3 Yale Department of Psychiatry, New Haven, CT USA; 4 Janssen Pharmaceuticals, San Diego, CA USA; 5 W.M. Keck Biotechnology Resource Laboratory, Yale School of Medicine, New Haven, CT USA; 6 CHU de Québec - Université Laval, Quebec City, Canada; 7 Department of Physiology Anatomy and Genetics, Oxford University, Oxford, UK; 8 Kavli Institute for Nanoscience Discovery, Oxford, UK

**Keywords:** Alzheimer's disease, Alzheimer's disease

## Abstract

Alzheimer’s disease (AD) is a complex and heterogeneous neurodegenerative disorder with contributions from multiple pathophysiological pathways. One of the long-recognized and important features of AD is disrupted cerebral glucose metabolism, but the underlying molecular basis remains unclear. In this study, unbiased mass spectrometry was used to survey CSF from a large clinical cohort, comparing patients who are either cognitively unimpaired (CU; n = 68), suffering from mild-cognitive impairment or dementia from AD (MCI-AD, n = 95; DEM-AD, n = 72), or other causes (MCI-other, n = 77; DEM-other, n = 23), or Normal Pressure Hydrocephalus (NPH, n = 57). The results revealed changes related to altered glucose metabolism. In particular, two glycolytic enzymes, pyruvate kinase (PKM) and aldolase A (ALDOA), were found to be upregulated in CSF from patients with AD compared to those with other neurological conditions. Increases in full-length PKM and ALDOA levels in CSF were confirmed with immunoblotting. Levels of these enzymes furthermore correlated negatively with CSF glucose in matching CSF samples. PKM levels were also found to be increased in AD in publicly available brain-tissue data. These results indicate that ALDOA and PKM may act as technically-robust potential biomarkers of glucose metabolism dysregulation in AD.

## Introduction

Alzheimer’s disease (AD) is the most prevalent form of dementia affecting over 6 million people in the United States in 2022^1^. The diagnostic pathological findings of AD in the brain are amyloid-beta (Aβ) plaques and paired helical filaments of hyperphosphorylated tau which reflect a disorder of proteostasis^2^. However, the causes and consequences of AD pathology are complex with varying degrees of inflammation^3^, neurovascular dysfunction^4^, and altered brain metabolism^5^ contributing to neurodegeneration and resultant dementia.

Multi-omic studies have suggested changes in several markers of glucose metabolism in both cerebrospinal fluid (CSF) and tissue from AD patients^6–8^. Moreover, a recent study of familial early-onset AD showed that elevation of glucose metabolism markers occurs early in AD disease progression, before elevation of inflammatory and neurodegenerative markers^9^. Recently, Traxler et al., (2022), reported a shift from aerobic to anaerobic glucose metabolism in an induced neuronal model of AD, indicating functional metabolic changes occurring within neurons in AD.

CSF is in continuous exchange with the interstitial fluid of the brain and is enriched with a host of proteins secreted, excreted, or otherwise released from neurons and glia. Thus, CSF remains the most accessible and informative matrix to measure biochemical changes occurring in the brain, whereas fluids like blood plasma struggle with low analyte concentration of neuronal and glial origin^10^.

Despite an identified association between altered brain glucose metabolism and AD, specific mechanistic biomarkers of altered glucose metabolism in AD have not been identified. Unbiased screening of protein and/or peptide levels in CSF, in relation to changes of the traditional Aβ and tau biomarkers in a clinically diverse cohort, could help identify distinct markers related to glucose metabolism that are altered in AD as opposed to other neurological processes.

Liquid chromatography coupled with tandem mass spectrometry (LC–MS) is a widely used and efficient method for the unbiased quantification of peptides and proteins in biofluids. Previous studies have used MS-based techniques to investigate the proteomic landscape of AD in both brain tissue and CSF^7,11–15^, highlighting multiple proteomic modules affected in AD, including energy metabolism. With technological advances over the last decade, data-independent acquisition MS (DIA-MS) methods can now be employed at a much larger scale, yielding increased depth of proteome coverage with high quantification accuracy^16–20^.

In this study, we performed DIA-MS on CSF collected during a patient's clinical evaluation in a neurology clinic, for suspected AD and a wide variety of other neurological disorders. Age matched cognitively unimpaired control samples were also obtained from clinic attendees. Utilizing a neurology clinic cohort, as opposed to a well-defined high-contrast cohort, more closely aligns with a real-life diagnostic situation, and demonstrates the potential applicability of our findings to a clinical setting.

The results show upregulation of multiple glycolytic enzymes in AD CSF. We highlight two key enzymes, PKM and ALDOA, as robust potential novel biomarkers for AD. We show that these enzymes are present as full-length proteins in CSF and that levels of these enzymes negatively correlate with CSF glucose levels, but not with a peripheral measure of long-term glucose dysregulation. Finally, we compared our CSF data to a well powered publicly available tissue dataset and showed that a similar elevation pattern of glycolytic enzymes in AD was observed in tissue. The data presented here expand upon previous findings of broad dysregulation of the glucose metabolism machinery in AD and highlight targets for further study.

## Results

### Cohort results

**Figure 1:**
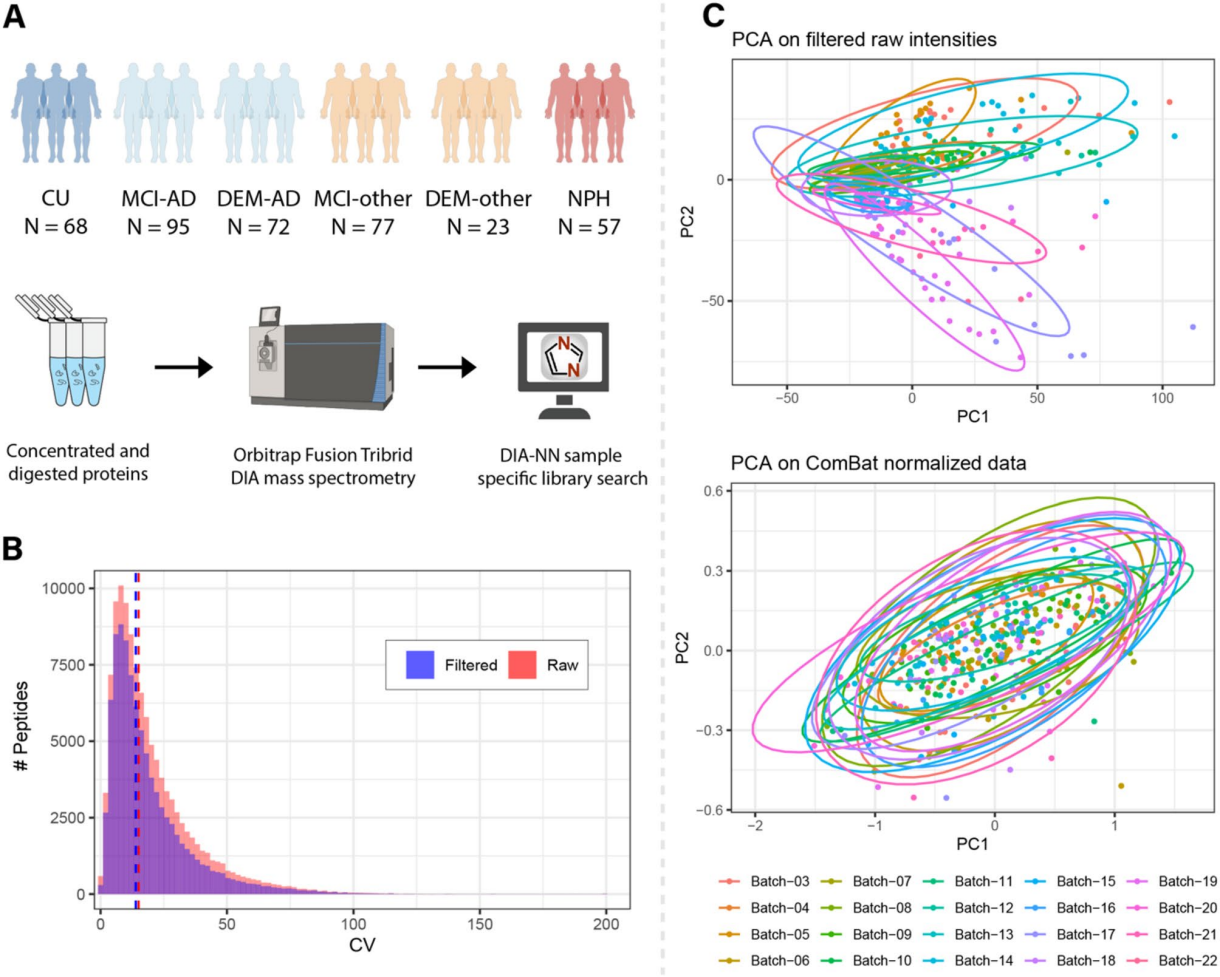
Experimental workflow and MS processing. (**A**) Overview of cohort numbers and a schematic overview of the sample processing. (**B**) Distribution of duplicate CV before (red) and after (blue) filtering of peptides that were measured in over 80% of all samples. After filtering the median duplicate CV dropped from 15.1 to 14.0%. (**C**) Principal component analyses before and after ComBat batch correction of the data. Dots represent individual samples and are colored by their corresponding batch. Before ComBat batch correction, a clear separation by batch can be observed in the first two principal components. This batch effect was mitigated after batch correction indicated by an overlap of the samples between batches.

The cohort analyzed by DIA-MS consisted of 400 different patient samples. The samples in this cohort reflected a diversity of patients from a neurology clinic spanning various non-dementia diagnoses and diagnoses of patients with cognitive impairments of varying degrees of severity. The cohort was selected to reflect a large age range (56–94 years). The samples in the AD groups were defined by a low Aβ42/40 ratio below the in-house determined threshold of 0.0818, to establish AD as the major pathophysiology for dementia, although mixed pathologies could not be ruled out. Nine CU samples were observed to be Aβ42/40 positive, reflecting "asymptomatic", "pre-clinical" or "AD resilient" status. These samples were included in the evaluation of the technical variability between injections and batches in MS but excluded from the downstream linear regression (Table 1, Fig. 1A).

**Table 1:** Clinical demographics.

Diagnosis	*N*	Age		Gender	Aβ42/40 ratio	pTau(181)	tTau
		Mean (SD)	Range	Females *N* (%)	Mean (SD)	Mean (SD)	Mean (SD)
CU	68	64.2 (7.53)	55–85	38 (55.9)	0.112 (0.015)	29.16 (10.61)	212.4 (74.2)
MCI-AD	95	70.7 (7.33)	56–86	44 (46.3)	0.052 (0.016) *,†,$	89.10 (46.55) *,†,#,$	442.6 (202.5)*,†,#,$
DEM-AD	72	71.5 (9.26)	56–93	33 (45.8)	0.053 (0.015)*,†,$	98.76 (50.91) *,†,#,$	470.3 (255.1)*,†,#,$
MCI-other	77	68.2 (7.59)	55–84	27 (35.1)	0.131 (0.197)	28.03 (18.48)	220.0 (115.1)
DEM-other	23	70.5 (9.51)	57–89	9 (39.1)	0.112 (0.023)	30.94 (21.10)	198.8 (89.8)
NPH	57	75.0 (7.73)	57–94	20 (35.1)	0.102 (0.027)	29.00 (21.66)	204.6 (114.1)

*CU =* Cognitively unimpaired and includes 20 with no other specified neurological disorders, 18 patients evaluated for immune disease, 9 patients with other non-dementing neurodegenerative diseases, 6 patients with vascular disease, 5 with demyelinating disease, 4 patients with headache, 3 patients with psychiatric disease, 3 patients with idiopathic intracranial hypertension, and 1 patient with neoplasm, *MCI-AD* = mild cognitive impairment due to Alzheimer’s disease; *DEM-AD* = dementia due to Alzheimer’s disease, *MCI-other* = mild cognitive impairment due to other non-AD causes, *DEM-other* = dementia due to other non-AD causes, *NPH* = normal pressure hydrocephalus. Only MCI-AD and DEM-AD subjects had CSF AD biomarkers with low Aβ42/40 indicating AD. All others had normal Aβ42/40 above diagnostic threshold value. Group differences were assessed with ANOVA and p-values indicate results from post-hoc Tukey test.

* = significantly different compared to CU (*p* < 0.05).

† = significantly different compared to MCI-other (*p* < 0.05).

# = significantly different compared to DEM-other (*p* < 0.05).

$ = significantly different compared to NPH (*p* < 0.05).

### Mass spectrometry search results

Peptide and protein level quantifications were obtained from DIA-NN. To establish a robust dataset, a series of quality control steps were completed. First, missing values that were observed in the dataset were filtered to only keep peptides that were identified and quantified in at least 80% of all samples (Supplementary Fig. 1). Following filtering, the duplicate coefficient of variation (CV) was calculated for pooled samples within each batch. Only the peptides with a mean duplicate CV below 25% were retained, resulting in a final dataset consisting of 4415 unique peptide sequences belonging to 636 unique proteins. By selecting these robustly quantified peptides, the median duplicate CV decreased from 15.1 to 14.0% (Fig. 1B). Remaining missing values were imputed using the mean intensity value for each peptide. Principal component analyses (PCA) revealed a batch-wise clustering of the samples (Fig. 1C), and the ComBat batch correction algorithm was applied to the dataset^21^. After ComBat batch correction one sample was observed as a clear outlier, leading to exclusion from downstream analysis. After this, the batch-wise clustering of samples was no longer observed in the PCA (Fig. 1C and data not shown).

### Differential abundance analysis.

To be inclusive of potential diagnostically relevant protein fragments or peptides^22,23^ in CSF, our initial data analysis was performed at the peptide level. After establishing a robust dataset of peptide level quantifications in CSF, differential expression of peptides across experimental groups was tested using a linear regression model. In total, there were 5452 significant contrasts between any two groups (adjusted *p* < 0.05). 3552 of these contrasts were between NPH and any group. The high number of contrasts that were derived from any group compared to NPH reflect a strong differential molecular phenotype of NPH compared to any of the other groups (Supplementary Fig. 2). For this reason, to further investigate the molecular differences between the AD and non-AD groups, differential expression in NPH was left out of consideration from subsequent analyses. This resulted in 1900 contrasts with an adjusted *p*-value below 0.05 between any non-NPH group, corresponding to 904 unique peptide sequences.

**Figure 2:**
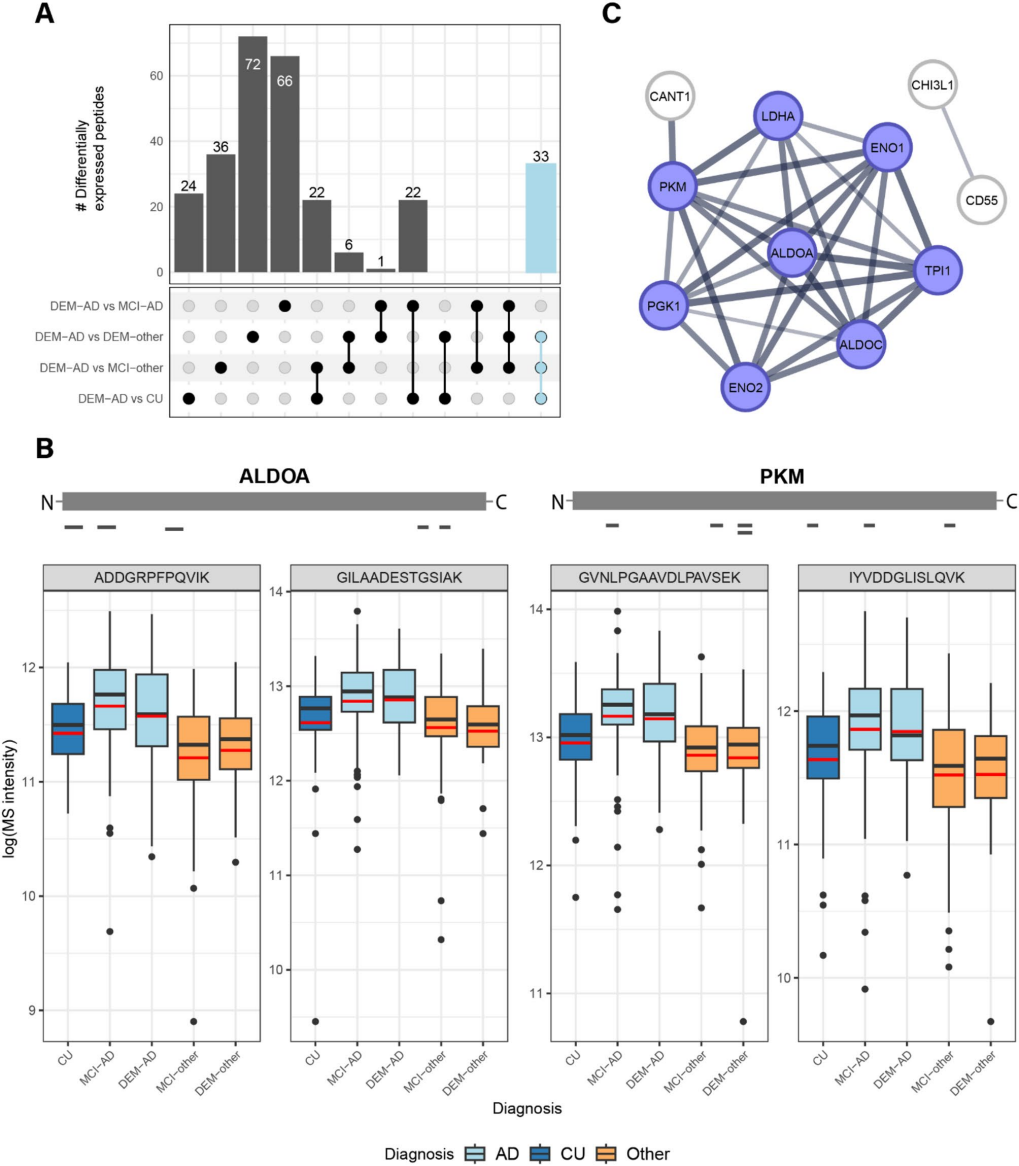
General upregulation of glycolytic enzymes is observed in AD CSF. (**A**) UpSet plot showing an overview of the differentially expressed peptides between DEM-AD and any non-AD group. The 33 peptides that were differentially abundant between DEM-AD and all non-AD group is highlighted in light blue. (**B**) Schematic representation of the peptide coverage of ALDOA and PKM in the MS data. Boxplots indicate the MS-intensity levels of 2 selected peptides from ALDOA and PKM each that were differentially expressed in an AD enriched pattern. Red lines indicate the mean. (**C**) Selected cluster from the STRING-DB analysis highlighting the enrichment of proteins involved in energy metabolism in AD CSF. Blue indicates proteins from the GO-term “canonical glycolysis”.

There were 399 contrasts between any diagnostic group compared to DEM-AD with an adjusted *p*-value < 0.05 (Fig. 2A, Supplementary Table 1). 33 peptides were differentially abundant between DEM-AD and all non-AD groups (CU, MCI-other and DEM-other). All these peptides were upregulated in DEM-AD compared to the non-AD groups. These peptides belong to the following 11 proteins: ALDOA, ALDOC, BASP1, ENO1, GDA, GOT1, LDHA, MDH1, NAXE, PKM and SMOC1 with various peptides belonging to each protein. Figure 2B highlights the peptide coverage and abundance of two peptides for both ALDOA and PKM. For both proteins, peptide coverage across the entire protein was observed.

### Protein interaction network

To further explore the proteomic differences, present in CSF in AD, a list of 120 proteins was assembled from the peptides that were found to be differentially abundant between DEM-AD and any other diagnostic group (Supplementary Table 1). This list was then used to perform a functional network analysis using STRING-DB^24^. A k-means clustering with seven clusters was applied on the network (Supplementary Fig. 3) and resulting clusters were functionally annotated with Gene Ontology (GO) biological process terms. One main cluster that was found to be annotated with GO terms for “glycolytic process” or “canonical glycolysis”, contained the proteins ALDOA, ALDOC, ENO1, ENO2, LDHA, PGK1, PKM and TPI1 (Fig. 2C). Other clusters were annotated with GO terms relating to neuronal signaling, immune response or lipid metabolism. The cluster annotated with immune response contained proteins such as C6, C9 and NPY. The neuronal signaling clusters contained multiple synaptic markers such as NPTX1, NPTX2 and NPTXR as well as VGF (nonacronymic), which were found to be downregulated in DEM-AD compared to CU and MCI-AD. The lipid metabolism cluster contained APOE, which is genetically associated to AD, and APP, the precursor that produces the peptides found in amyloid plaques. One APOE peptide was downregulated in DEM-AD compared to CU, whereas one APP peptide was upregulated in DEM-AD compared to MCI-other.

### Determining the presence of full-length protein in CSF

**Figure 3:**
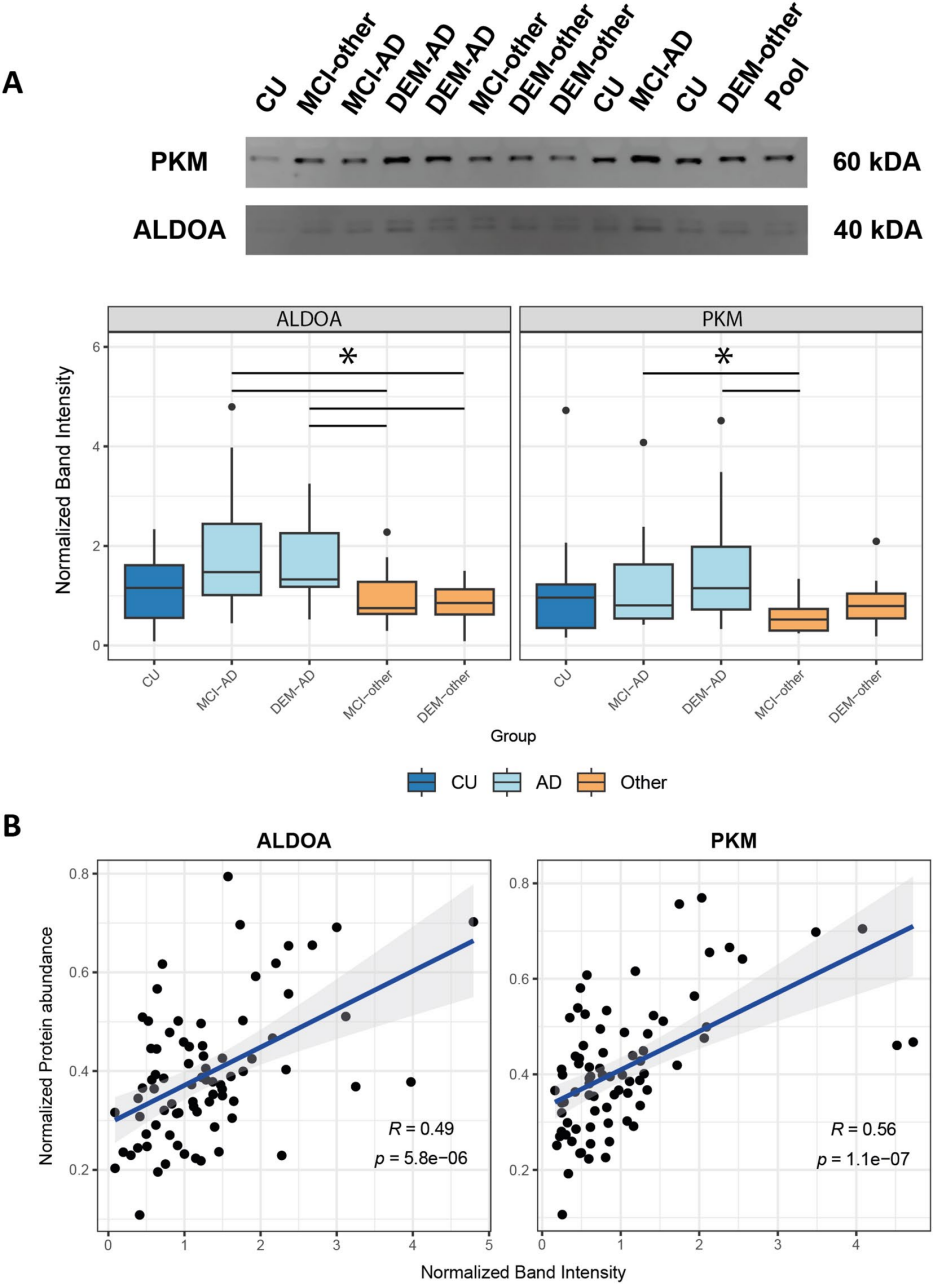
Immunoblotting of ALDOA and PKM indicate the presence of full-length proteins in CSF. (**A**) Western blot bands at the expected sizes of 60 kDa for PKM and at 40 kDa for ALDOA indicate the presence full length protein. Normalized band intensities of ALDOA and PKM follow similar AD enrichment pattern across groups as the protein level quantifications for ALDOA and PKM (*: Post-hoc Dunn test *p* < 0.05). Original blots are presented in Supplementary Fig. 4A. (**B**) Protein level MS-intensity of both ALDOA and PKM are significantly correlated with the normalized western blot band intensity. This positive correlation indicates the presence of full-length ALDOA and PKM proteins in CSF.

With the upregulation of multiple peptides for various glycolytic enzymes in DEM-AD, we aimed to confirm these peptides were derived from full-length proteins, present in CSF, through immunoblotting. A balanced subset of the samples that were used for MS was randomly selected to verify the MS peptide level quantifications (Supplementary Table 4). Two of the main glycolytic enzymes that were found to have multiple peptides that were specifically elevated in AD compared to other experimental groups, ALDOA and PKM, were assessed by immunoblot for protein level quantifications. Expected full-length protein sizes of 40 kDa and 60 kDa for ALDOA and PKM, respectively, was confirmed using recombinant protein (Supplementary Fig. 4B). A double banded pattern was detected for ALDOA at the expected 40 kDa size, suggesting the presence of two variants of full-length ALDOA in CSF. For PKM, a single band was detected at the expected 60 kDa size indicating the presence of full-length protein in CSF (Fig. 3A).

Relative band intensity compared to a pooled sample was measured across diagnoses for all targets (Fig. 3A; Supplementary Fig. 4A). For both ALDOA and PKM, the normalized band intensities followed a similar abundance pattern to their tryptic peptides in MS and non-parametric Kruskall–Wallis tests indicated significant differences between groups. For ALDOA, post-hoc Dunn-test showed that both MCI-AD and DEM-AD band intensities were significantly increased compared to both MCI-other and DEM-other (*p* < 0.05). PKM normalized band intensities were also significantly elevated in both MCI-AD and DEM-AD compared to MCI-other (*p* < 0.05).

To further establish that the measured MS-intensities for these markers reflected the levels of full-length proteins in CSF, correlations between normalized band intensity and the protein-level MS-intensity for the glycolytic enzymes ALDOA and PKM were determined (Fig. 3B). For PKM a significant positive correlation was observed (r = 0.56, *p* = 1.1 × 10^–7^). For ALDOA a significant positive correlation was observed (r = 0.49, *p* = 5.8 × 10^–6^).

### Changes in metabolic markers in CSF and periphery

**Figure 4:**
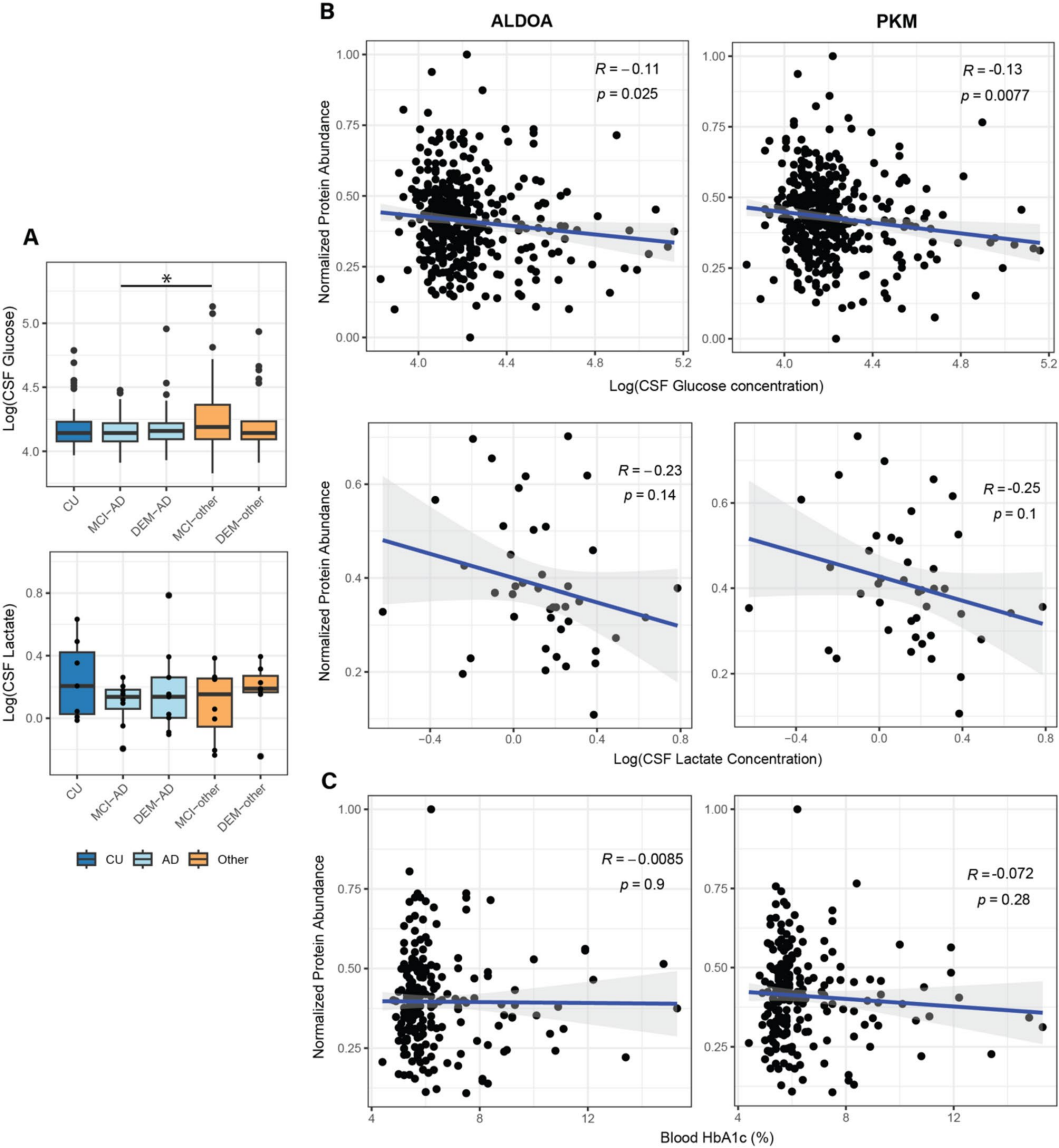
Levels of glucose, lactate and HbA1c in CSF in relation to glycolytic enzymes. (**A**) Levels of glucose and lactate in CSF across groups. CSF glucose levels were only found to be downregulated in MCI-AD compared to MCI-other. CSF lactate levels were not found to be significantly differentially abundant between any diagnostic group (*: Pearson’s correlation *p* < 0.05). (**B**) Protein level MS intensities for ALDOA and PKM showed a significant negative correlation with the CSF glucose levels. A negative trend was observed between CSF lactate and ALDOA and PKM protein abundance, but this trend did not reach significance. (**C**) No correlation was observed between peripheral glucose metabolism marker HbA1c and MS intensities for ALDOA and PKM proteins.

Following the potential indication of altered glucose metabolism in the CSF of AD patients, the levels of selected metabolites in the same CSF samples were investigated across the whole cohort. In a group level comparison, CSF glucose levels were only elevated in MCI-other compared to MCI-AD (*p* < 0.05), but not in any other contrast. In a balanced subset of the cohort (Supplementary Table 4), no significant changes in lactate levels were observed between groups (Fig. 4A). CSF glucose levels were found to be significantly negatively correlated with the protein-level MS-intensity of both ALDOA (r = − 0.11, *p* = 0.025) and PKM (r = − 0.13, *p* = 7.7 × 10^–3^). Both MS protein abundances of ALDOA and PKM showed a negative trend with CSF lactate levels, but these trends did not reach significance (*p* > 0.05) (Fig. 4B). Hemoglobin A1C (HbA1c) values, a measure of long-term peripheral glucose dysregulation were obtained from clinical records for 207 individuals (Supplementary Table 4). Neither ALDOA nor PKM showed a significant correlation with HbA1C (Fig. 4C).

### Levels of glycolysis markers in CSF and tissue

**Figure 5:**
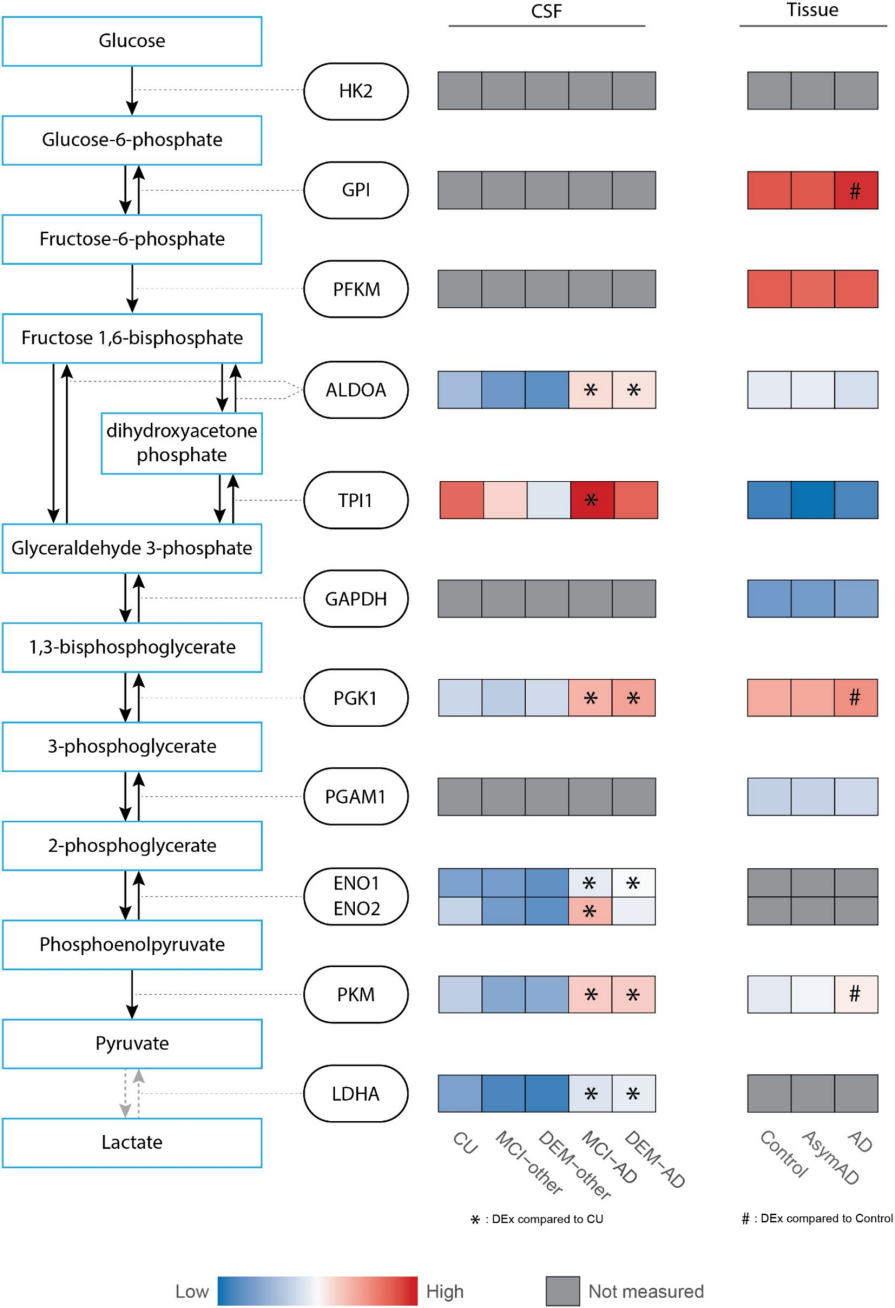
General elevation of glycolytic enzymes in AD is observed in both CSF and brain tissue. Heatmap representation of protein abundance of glycolytic enzymes measured in our CSF dataset and from a publicly available tissue dataset. Levels of ALDOA, PGK1, ENO1, PKM and LDHA were significantly upregulated in DEM-AD compared to CU in CSF. In tissue, GPI, PGK1 and PKM were significantly upregulated in AD compared to Controls (*: post hoc Tukey test *p* < 0.05). Protein abundances were scaled within each dataset and colors represent relative protein abundance. Gray boxes indicate proteins that were not robustly quantified in CSF or tissue.

With the peptide level enrichment of the two key glycolytic enzymes ALDOA and PKM in DEM-AD, alongside an indication of general glycolytic dysregulation from STRING analysis, we investigated protein level changes in the levels of all glycolytic enzymes using the same linear regression model used for the peptide level. The first three enzymes in glycolysis, HK2, GPI and PFK1, along with GAPDH and PGAM1 were not robustly measured in CSF in this analysis. The other enzymes, ALDOA, PGK1, ENO1, and LDHA were all significantly upregulated in DEM-AD compared to CU, MCI-other and DEM-other (adjusted *p*-value < 0.05; Fig. 5). TPI1 was significantly upregulated in DEM-AD compared to MCI-other and DEM-other but not CU (adjusted *p*-value < 0.05). ENO2 was significantly upregulated in DEM-AD compared to MCI-other. Additionally, all proteins were significantly upregulated in MCI-AD compared to CU, MCI-other and DEM-other, with the exception of PGK1 (Supplementary Table 3).

To gain insight into the possible source of the changes of glycolytic enzymes in CSF, publicly available protein abundance data from brain tissue was investigated^7^. Here, AD brain tissue from the dorsolateral prefrontal cortex was compared to tissue from healthy controls and subjects without dementia but with postmortem AD pathology (AsymAD). The mean scaled abundances for each group were plotted and significance was annotated using the author’s significance values generated from post hoc Tukey tests (Fig. 5). The enzymes, GPI, PGK1 and PKM were elevated in AD compared to controls (*p* < 0.05). GPI, PGK1 and TPI1 were elevated in AD compared to AsymAD (*p* < 0.05), suggesting their elevation is related to cognitive dysfunction as opposed to amyloid pathology. HK2, ENO1 and ENO2, and LDHA were not robustly measured in brain tissue, as defined by being quantified in less than 50% of all samples. Although the changes of specific glycolytic enzymes do not necessarily correspond between tissue and CSF, a general elevation of multiple glycolytic enzymes in both matrices indicate general disturbance of glycolysis in the AD brain.

## Discussion

Heterogeneity amongst AD patients necessitates the stratification of patients to specific molecular targets for effective therapeutic intervention. The complex and heterogeneous nature of AD and its intersecting biological pathways have made it difficult to specify these targets. Here, we present a deep CSF proteomic survey of a large clinically diverse patient cohort. Differential expression indicated a strong enrichment of glucose metabolism markers in AD compared to non-AD groups. Specifically, the glycolytic enzymes ALDOA, ALDOC, ENO1, LDHA and PKM were found to be elevated following an upregulated expression pattern in DEM-AD compared to all non-AD groups. Through immunoblotting, both ALDOA and PKM were verified to be present as full-length proteins in CSF. Protein level quantifications from these glycolytic enzymes negatively correlated with glucose levels and showed a negative trend with lactate levels in the corresponding CSF sample. Finally, we showed that other glycolytic enzymes follow a similar pattern of enrichment in AD in CSF and brain tissue homogenate, although there are sample type specific effects.

Glucose is the primary fuel that powers the vast synaptic activity in the human brain and is measured by fluorodeoxyglucose positron emission tomography (FDG-PET)^25–27^. Decreases in cerebral glucose consumption, measured with FDG-PET correlate strongly with decreased levels of the pre-synaptic protein synaptophysin in post-mortem tissue^28^, and is a central biomarker of disease^27,29, 30^. Neurons require efficient metabolism of glucose through glycolysis and oxidative phosphorylation^26,31^. Alternatively, in events of brain injury, lactate can be used as an alternative source of energy^26,32^. Evidence of neuronal insulin resistance, as measured in postmortem tissue research, is also well described^33–35^, with one of the major risk factors for AD being type 2 diabetes mellitus, a disease characterized by insulin resistance. Previous studies demonstrated changes in proteins involved in energy metabolism in CSF from patients with AD; specifically, both PKM and ALDOA were upregulated in patients with AD dementia^6,7, 9^. Our findings validate these reports and indicate, through immunoblotting, that both ALDOA and PKM are present as full-length proteins in CSF. Recently published data has also shown that pyruvate kinase is present in CSF as a functional protein and its activity is increased in AD highlighting its potential as a mechanistic biomarker of dysregulated glucose metabolism^36^. This study used an enzymatic assay that was not able to distinguish between isoforms of pyruvate kinase, but it would make intuitive sense that the elevation of activity that they observe correlates with the increase in PKM levels that we observe. Altered glucose metabolism in the brain has long been observed as a key pathophysiological feature of AD. Brain hypometabolism as measured by FDG-PET indicates a decreased uptake of glucose into the cell through the glucose transporters GLUT1 and GLUT3^37^. Reduced cerebrovascular blood flow in AD can lead to hypoxic conditions in the brain^38^, which induces an upregulation of hypoxia induced factor 1 (HIF1)^39^. This transcription factor directly upregulates the transcription of GLUT1 and GLUT3 as well as multiple glycolytic enzymes, inducing glycolytic metabolism^39^. The glycolytic enzymes, ALDOA and PKM, presented here may be reactive markers of this process in CSF.

PKM is a critical rate-limiting enzyme in glycolysis that regulates the generation of pyruvate, and as such it has been widely studied in cancer cells, a cell type with high metabolic activity^40^. Aerobic glycolysis occurs when pyruvate is processed through the tricarboxylic cycle, resulting in a high energy yield. By contrast, tumor cells anaerobically convert pyruvate into lactate via LDHA in a phenomenon termed the Warburg effect^40^. The changes in neuroenergetics occurring in AD have previously been described as an inverse Warburg effect^41,42^. In an AD patient-derived induced neuronal model, it was shown that one specific PKM isoform, PKM2, regulates an anaerobic glycolytic shift, similar to the Warburg effect, whereas PKM1 regulates aerobic glycolysis^43^. A PKM2-specific targeting anti-cancer drug was shown to ameliorate this effect. PKM2, has also been directly linked to Ab plaque promotion by positively regulating gamma-secretase in the cytosol in hypoxic conditions^44^. The peptides identified by DIA in this study could not distinguish between the PKM1 and PKM2 isoforms, and immunoblotting with a PKM2 specific antibody in CSF samples showed no quantifiable signal (data not shown). However, the results presented here potentially indicate that the changes in PKM levels we observe in patient-derived CSF may be related to the changes described by Traxler et al^43^ who show widespread increases in most glycolytic enzymes in response to elevation of metabolically inactive PKM2.

ALDOA has previously been associated with AD and other neurological disorders^45,46^, including its identification as a possible autoantigen in AD^46^. While overlapping pathology between AD and other groups suggested ALDOA was a non-specific biomarker for AD and thus a poor candidate for immunotherapy^45,47^, the utility of ALDOA as a biomarker for stratification within defined AD patients has not been fully explored.

In this study, both ALDOA and PKM were observed to be significantly negatively correlated with CSF glucose levels and followed a negative trend with CSF lactate levels. Glucose and lactate levels in CSF have been studied before as markers of glucose metabolism dysregulation in AD, however the direction of changes have remained inconclusive^48–51^. Higher CSF glucose levels have been found to be associated with less tau pathology in the brain^48^. A reduction in CSF lactate has been observed in AD which was suggested to be linked to tauopathy^49^. In our data, we observe that increased abundance of glycolytic enzymes is correlated with lower CSF glucose, potentially indicating an increased cellular uptake of glucose through upregulated glucose transporters. One result of increased glycolysis is the increase in production of lactate, which can be transported out of the cell through monocarboxylate transporter 2 (MCT2)^52^. We report no significant correlation between abundance of glycolytic enzymes and CSF lactate, but a negative trend was observed. Lactate is known to channel between different cells and cell types through lactate shuttles^52^, indicating increased lactate might be taken up by glial cells instead of being released into CSF, providing a potential explanation why lactate is not increased in CSF in relation to upregulated glycolytic enzymes.

This leads to the question how the glycolytic enzymes are upregulated in CSF. Potentially, these increases comes from the neurodegenerative process occurring in the AD brain where the enzymes are simply released into the CSF upon cell death. Alternatively, an in-vitro model of iPSC-derived choroid plexus cells that produce CSF has been shown to release glycolytic enzymes into the extracellular space^53^. Conceivably, these cells also experience a HIF1 induced upregulation of glycolytic metabolism through hypoxic stress, subsequently leading to an increased release of these enzymes into the CSF. However, mechanistic confirmation remains necessary to address this.

### Limitations

Despite the technological advances in DIA-MS that allow for experiments on large numbers of samples, the downstream analysis of such experiments is still a subject of ongoing development. Analyses on a large-scale cohort, as presented here, require a batch-wise division in MS scanning. Advances in batch-correction methods specifically developed for MS data is expected to improve the analyses of such large-scale datasets^54,55^. To overcome these issues in this study, very robust cutoffs were applied when curating the dataset, resulting in high confidence on the identification and quantitation of the peptides and proteins presented here. However, this could implicate that some informative peptides and proteins were excluded from our analyses. Furthermore, this analysis only included peptides originating from 2 tryptic cleavages. Peptides derived from non-tryptic cleavages have been shown to be present and active in the brain and CSF^56^. Expanding the peptide identification search to include semi-tryptic peptides that include only one tryptic cleavage at the N-terminal or C-terminal site in CSF would create a more representative picture of the endogenous peptide and fragment landscape^57,58^ but the expansion of the search space and need for FDR correction of peptide identifications generally means computational detection of these peptides is limited, and it is best to perform an experiment which enriches specifically for non-tryptic peptides^56,59^.

The publicly available tissue data used here only included data on the DLPFC. Johnson et al.^7^ showed that the changes of protein modules they observed to be upregulated in AD in the DLPFC were highly conserved in other brain regions they studied, like the temporal cortex and the precuneus. However, it would still be interesting to see how the levels of these enzymes behave across various brain regions affected at different Braak stages by pathology in AD.

Although the large sample in this study yields a better understanding of a real-world clinically diverse cohort, it is limited by the absence of longitudinal cognitive data and samples. Measurements of better biomarkers over time are imperative to better describe disease progression from early stages to further developed disease^60^.

In conclusion, this study applied DIA-MS on CSF from a large-scale cohort, spanning a variety of neurological and dementia related diagnoses, confirming previous findings of dysregulated glucose metabolism in AD. We find an enrichment of two glycolytic enzymes, ALDOA and PKM, in AD and highlight these enzymes as putative biomarkers for impaired brain metabolism in the AD brain.

## Methods

### Study cohort

CSF samples were obtained according to standardized collection and processing protocols through the Massachusetts General Institute for Neurodegenerative Disease biorepository, following written informed consent for research biobanking (IRB: 2015P000221). All methods were performed in accordance with the ethical standards of the Declaration of Helsinki. This repository consists of CSF samples from diagnostic lumbar punctures at the Department of Neurology at Massachusetts General Hospital, with the inclusion criterion for this study of anyone over the age of 55. CSF levels of Aβ_1–40_, Aβ_1–42_, pTau (181) and total tau (tTau) were measured by commercially available ELISA assays (Euroimmun, Lubeck, Germany). Clinical diagnoses were established through review of all available clinical data (including symptom history, diagnoses of treating neurologist, laboratory data, neuroimaging and neuropsychological testing, as available) by an experienced neurologist (SEA) and AD status was corroborated with CSF AD biomarkers showing Aβ_42/40_ ratio below the in-house threshold of 0.0818. At this ratio sensitivity is 91.6% and specificity is 91.3%. Samples were subdivided into six groups based on clinical diagnosis: cognitively unimpaired (CU; n = 68), mild cognitive impairment due to AD (MCI-AD; n = 95), mild cognitive impairment due to other causes (MCI-other; n = 77), dementia due to AD (DEM-AD; n = 72), dementia due to other causes (DEM-other; n = 23) and normal pressure hydrocephalus (NPH, based on positive “tap-test” gait outcome after large volume lumbar puncture; n = 57) (Table 1). Cognitively unimpaired individuals with CSF Aβ positivity (n = 9) were excluded from the study.

### CSF processing

Samples were processed for LC–MS/MS by investigators blinded to clinical status. Aliquots were frozen and stored at − 80 °C in low-binding polypropylene tubes and thawed on ice for use. 200 μL samples, when possible, were dried in a SpeedVac, and resolubilized in 100 μL 8 M urea/0.4 M ammonium bicarbonate, or half the original volume for low volume aliquots. Protein concentration was measured through Pierce BCA assay (Thermo Fisher Scientific, Waltham MA, USA) and samples were adjusted to 7.5 µg protein in 50 µL. Samples were reduced with a 1:10 dilution of 45 mM DTT and incubated at 37 °C for 30 min. Subsequently, samples were alkylated with a 1:10 dilution of 100 mM iodoacetamide at room temperature in the dark for 30 min. Urea concentration was then lowered to 2 M using water. Proteins were digested overnight at 37 °C with 1:20 LysC:sample protein followed by an additional 1:20 trypsin digestion for 8 h at 37 °C. Samples were then acidified with 20% TFA and desalted using Nest Group C18 macrospin columns (HMMS18V) following the manufacturer’s instructions, and the eluent was dried for mass spectrometry.

### Data-independent acquisition

DIA LC–MS was performed using a nanoACQUITY UPLC system (Waters Corporation, Milford, MA, USA) connected to a Thermo Orbitrap Fusion mass spectrometer (ThermoFisher Scientific, San Jose, CA, USA). Samples were resuspended in dH_2_O with 2% acetonitrile and 0.2% TFA and injected across 22 batches of 24 samples at a time, with each sample injected in duplicate and a pooled control sample at the start and end of each batch. After injection, the samples were loaded into a trapping column (Waters ACQUITY UPLC M-Class Symmetry® C18 trap column, 5 micro, 180 μm × 20 mm) at a flow rate of 5 µL/min and separated with an analytical column (Waters ACQUITY UPLC M-Class Peptide BEH C18 column, 1.7 micro, 75 µm × 250 mm). The compositions of mobile phases A and B were 0.1% formic acid in water and 0.1% formic acid in acetonitrile, respectively. The peptides were separated and eluted with a 120-min gradient extending from 6 to 35% mobile phase B in 85 min and then to 85% mobile phase B in an additional 5 min at a flow rate of 300 nL/min and a column temperature of 37 °C. Column regeneration and up to three blank injections were carried out in between all sample injections. The data were acquired with the mass spectrometer operating in a Data-Independent Acquisition mode with an isolation window width of 25 m/z. The full scan was performed in the range of 400–1000 m/z with “Use Quadrupole Isolation” enabled at an Orbitrap resolution of 120,000 at 200 m/z and automatic gain control (AGC) target value of 4 × 10^5^. Fragment ions from each MS^2^ non-overlapping isolation window were generated in the C-trap with higher-energy collision dissociation at a collision energy of 28% and detected in the Orbitrap at a resolution of 60,000. Gas phase fractionated DIA (GPF-DIA) acquisitions were collected by six injections of digested pooled samples from 400 to 500 m/z, 500 to 600 m/z, 600 to 700 m/z, 700 to 800 m/z, 800 to 900 m/z, and 900 to 1000 m/z with 4 m/z-wide windows at 100 ms injection time. For MS1 scan, the resolution was set as 120 K, AGC target value of 4 × 10^5^. For DIA scan, the resolution was set as 60 K, AGC target value of 1 × 10^5^ and precursor ions were fragmented with higher-energy C-trap dissociation (HCD) of 28%.

Raw files were processed with DIA-NN software (version 1.8.1) for peptide and protein identification and quantification^61^. DIA-NN was used in two steps as described in the DIA-NN manual (https://github.com/vdemichev/DiaNN). First, a library-free search on the GPF files using the Uniprot Reference Homo Sapiens database (UP000005640, 80581 sequences, 14.10.2022) was used to generate an *in-silico* library. Second, a library-based search was carried out on the raw files using the spectral library generated in step one. Search parameters were set as follows: Protease: Trypsin/P; Missed cleavages: (1) N-term excision of methionine enabled; Maximum number of variable modifications: (2) Variable modification: methionine oxidation; Fixed modification: cysteine carbamidomethylation; Peptide length range: 7–30 amino-acids; Precursor charge range: 2–4. Precursor and fragment ion m/z ranges were set according to acquisition parameters. Match between runs (MBR) was enabled only for the second step. All the other parameters were set as default value. To obtain normalized peptide and protein tables from the DIA-NN output, the main report was used with the DIA-NN R Package (https://github.com/vdemichev/diann-rpackage) and *peptides.maxlfq* and *protein.groups* tables were generated. Both are normalized with the MaxLFQ algorithm and filtered at 1% FDR at precursor and protein group levels^62^.

### Immunoblotting

A random balanced subset of samples from the mass spectrometry cohort was selected as a sub cohort (Supplementary Table 4). Samples were spun down at 3000 × g for 10 min and the resulting supernatant was used for immunoblotting. Total sample protein concentration was determined using the Pierce BCA assay and 20 µg of protein per sample was loaded on the gel. A pooled sample was generated from the samples and loaded on each gel for between-blot normalization. Novex Wedgewell 4–20% polyacrylamide gels (Invitrogen, Waltham MA, USA) were used, and samples were run at 55 mA per gel. Subsequently, samples were transferred to nitrocellulose membrane at 300 mA for 90 min (Bio-Rad laboratories, Hercules CA, USA). After transfer, membranes were blocked for 1 h using LI-COR PBS intercept blocking buffer. Primary antibody dilutions were prepared using the LI-COR Intercept T20 PBS antibody diluent. Antibodies were used for ALDOA (D73H4, Cell Signaling Technology) at a concentration of 1:500, and PKM (C103A3, Cell Signaling Technology) at 1:1000. Membranes were incubated overnight in primary antibody at 4 °C. Membranes were then washed 4 × in PBS-T and incubated for 1 h in LI-COR goat anti-rabbit secondary antibody diluted 1:1000 in LI-COR Intercept T20 PBS antibody diluent. Blots were imaged with a LI-COR CLx imager and band intensities compared to background were measured using the Image Studio Light and ImageJ. High sensitivity ECL immunoblotting was performed by stripping the blot from previous antibodies with *OneMinute®Plus* Western Blot Stripping Buffer according to manufacturer’s protocol (GM Biosciences). Then the blot was blocked for 1 h with LI-COR PBS intercept blocking buffer and incubated overnight at 4 °C with primary antibody for ALDOA at 1:500 in LI-COR Intercept T20 PBS antibody diluent. Secondary HRP-conjugated antibody was incubated for 2 h at 1:10.000 in LI-COR Intercept T20 PBS antibody diluent. Enhanced chemiluminescence was performed with SuperSignal West Femto Maximum Sensitivity Substrate (ThermoFisher Scientific) according to manufacturer’s protocol, and blot was imaged on film.

### CSF glucose lactate and HbA1c measurement

CSF glucose levels were assessed in the Pathology Core at Massachusetts General Hospital through clinical standard enzymatic hexokinase assay at the same time as the CSF collection. Lactate concentration was measured on a randomly selected balanced subset of matched samples (Supplementary Table 4) through enzymatic assay (ab65331, Abcam), according to the manufacturer’s protocol. Samples were diluted 12 × to fall within the detectable range. Total glucose and lactate levels between diagnostic groups was determined and concentrations were correlated to the MS protein intensity of ALDOA and PKM. HbA1c measurements were obtained through electronic health records from matching patients where available. For patients with multiple HbA1c measurements, the highest value was used for analysis.

### Statistical analysis

All data analysis was performed in R-studio under R version 4.2.2. Raw MS intensities were imported from DIA-NN and processed as described in results. Pattern of missingness was determined by counting the number of samples per peptide where that peptide was not quantified. Batch correction was performed using the *ComBat()* function from the sva package (version 3.46.0). Linear modeling of the MS data was performed using the *lm()* function adjusting for age and sex as covariates: *lm(peptide intensity* ~ *experimental group* + *age* + *sex).* Results were tidied up using the *tidy()* function from the broom package (version 1.0.4) and *p*-values were corrected for multiple testing using the Benjamini–Hochberg method. Correlations were calculated with Pearson correlation methods using the *cor.test()* function. Between group differences for immunoblotting and metabolite levels were calculated using non-parametric Kruskall–Wallis test using *Kruskal.test()* and post-hoc analyzed with *dunnTest()* from the FSA package (version 0.9.4). The analysis of publicly available tissue data was done by plotting the mean scaled abundances for each group, and significance was annotated using the author’s significance values generated from post hoc Tukey tests.

### Study approval

This study was approved by the Institutional Review Board of Mass General Brigham (IRB 2015P000221) and all study participants or their assigned surrogate decision makers had provided written informed consent for use of their samples in biomarker research. All methods were performed in accordance with the ethical standards of the Declaration of Helsinki.

### Supplementary Information

Supplementary Figures.

Supplementary Tables.

## Data Availability

Normalized MS intensity values of all glycolytic proteins can be found in supplementary table 2. Underlying data has been deposited to ProteomeXchange through MassIVE (PXD043216).
