# Supplementary material for: Mass spectrometry in cerebrospinal fluid uncovers association of glycolysis biomarkers with Alzheimer’s disease in a large clinical sample
Source: Sci Rep. 2023 Dec 16;13:22406. doi: 10.1038/s41598-023-49440-3 (PMC10725469; doi:10.1038/s41598-023-49440-3)
Supplement: Supplementary file 1 — Supplementary Figures. [file 41598_2023_49440_MOESM1_ESM.pdf]

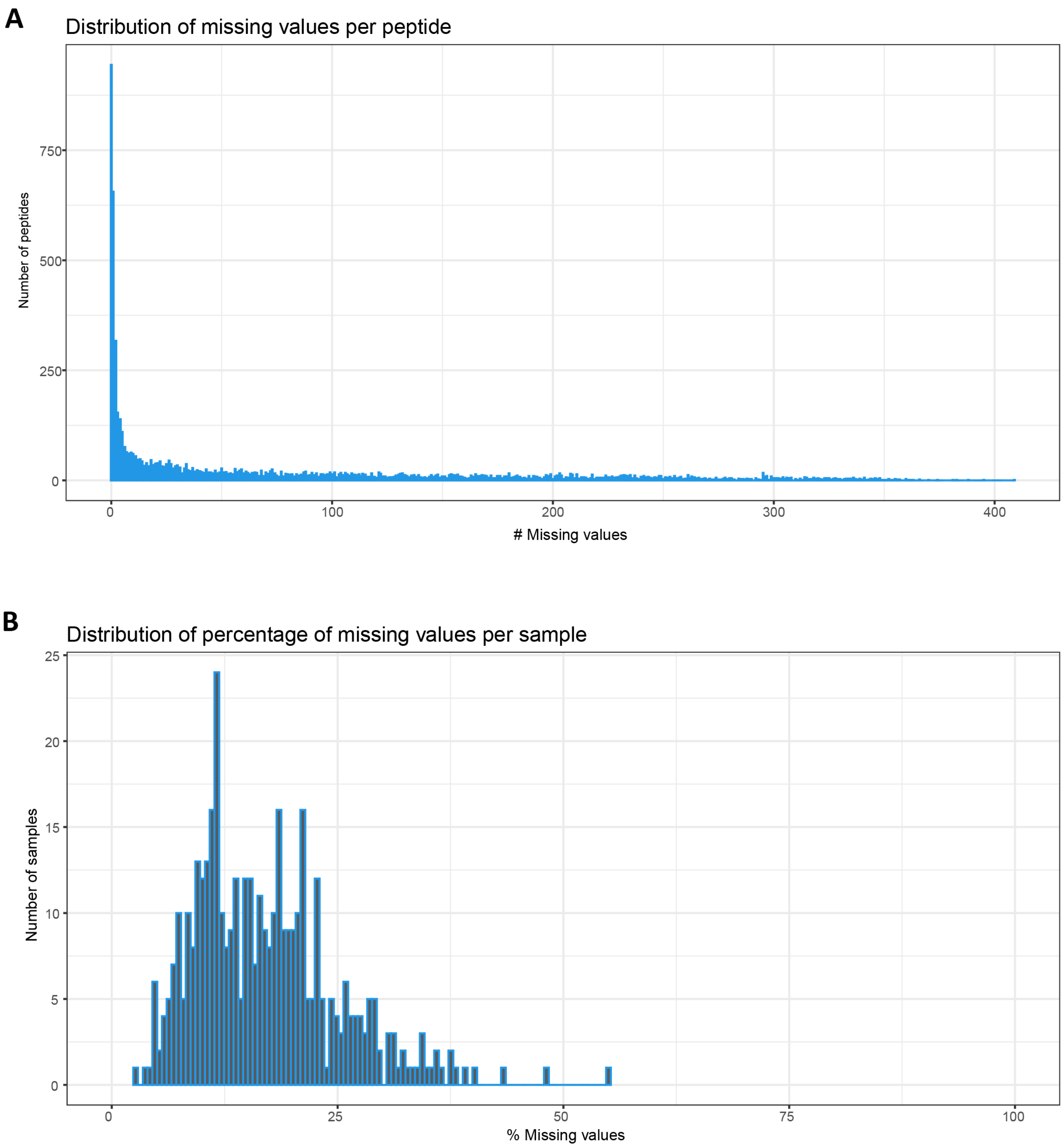

Supplementary Figure 1: A*) Distribution of the missingness for all peptides across the dataset. There were 944 peptides that had no missing values and the median number of missing values was 25. Peptides were filtered for expression in at least 80% of all samples, resulting in a dataset comprised of 4415 unique peptide sequences corresponding to 636 unique protein groups.* ***B****) Distribution of the percentage of missing values per sample. The median percentage of missing values per sample was 16%.*

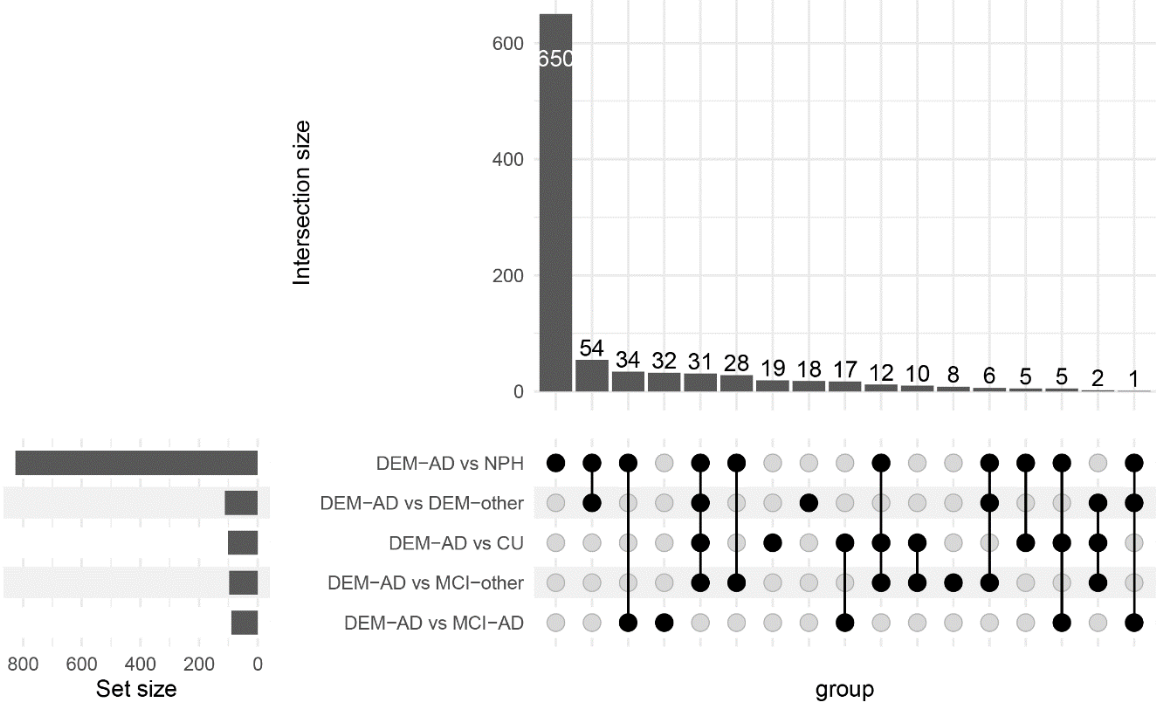

Supplementary Figure 2: *Overview of the differentially abundant peptides between DEM-AD and all other groups including NPH. There were 650 peptides uniquely differentially abundant between DEM-AD and NPH indicating a strong differential molecular phenotype of NPH.*

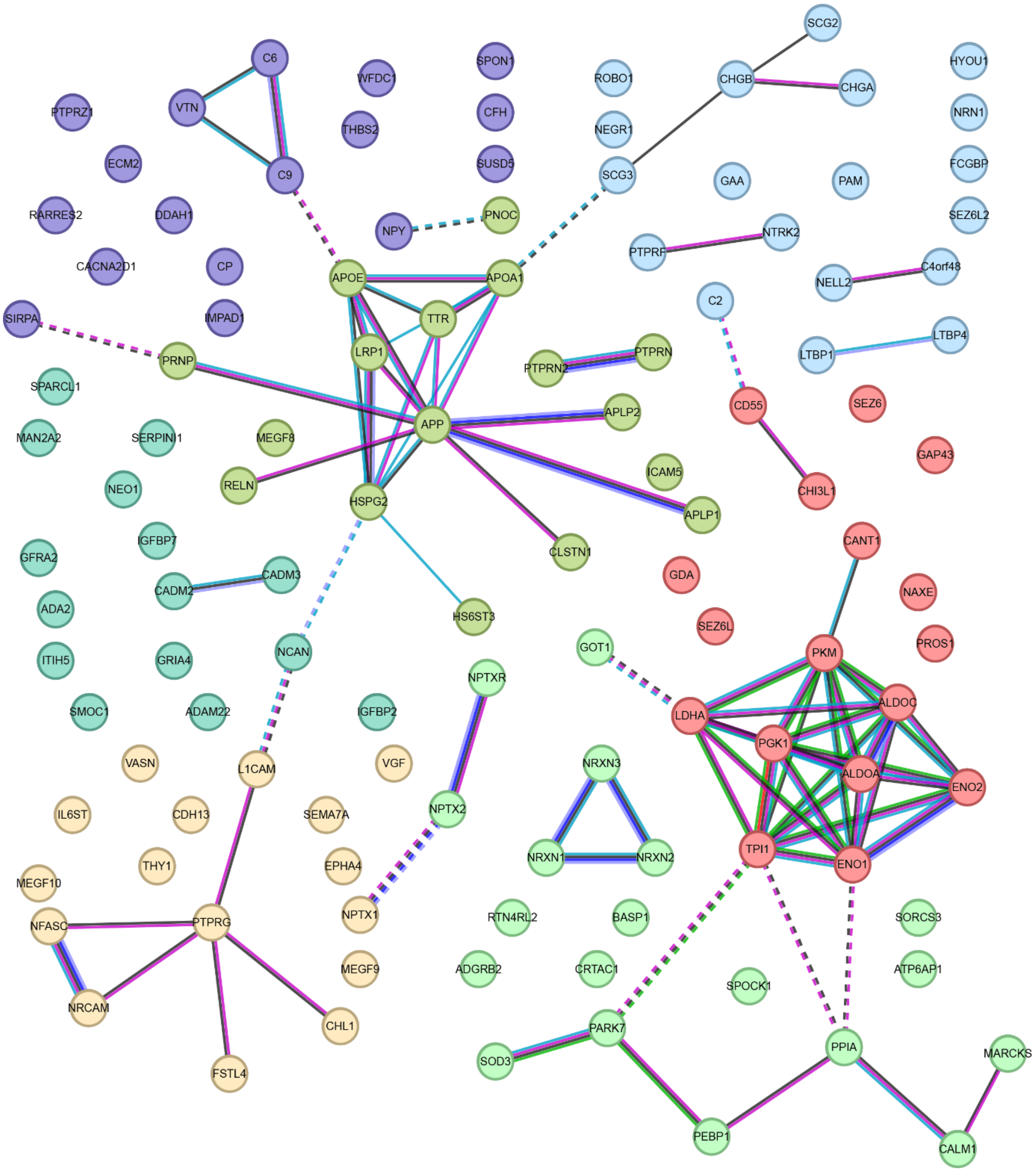

Supplementary Figure 3: *The complete STRING-DB functional network analysis output. 120 proteins that were found to be differentially abundant between DEM-AD and any other diagnostic group were used as input and a k-means clustering of 7 clusters was applied. Among the resulting clusters that were highly connected there was representation of GO terms for "canonical glycolysis", "neuronal signalling", "immune response" and "lipid metabolism.*

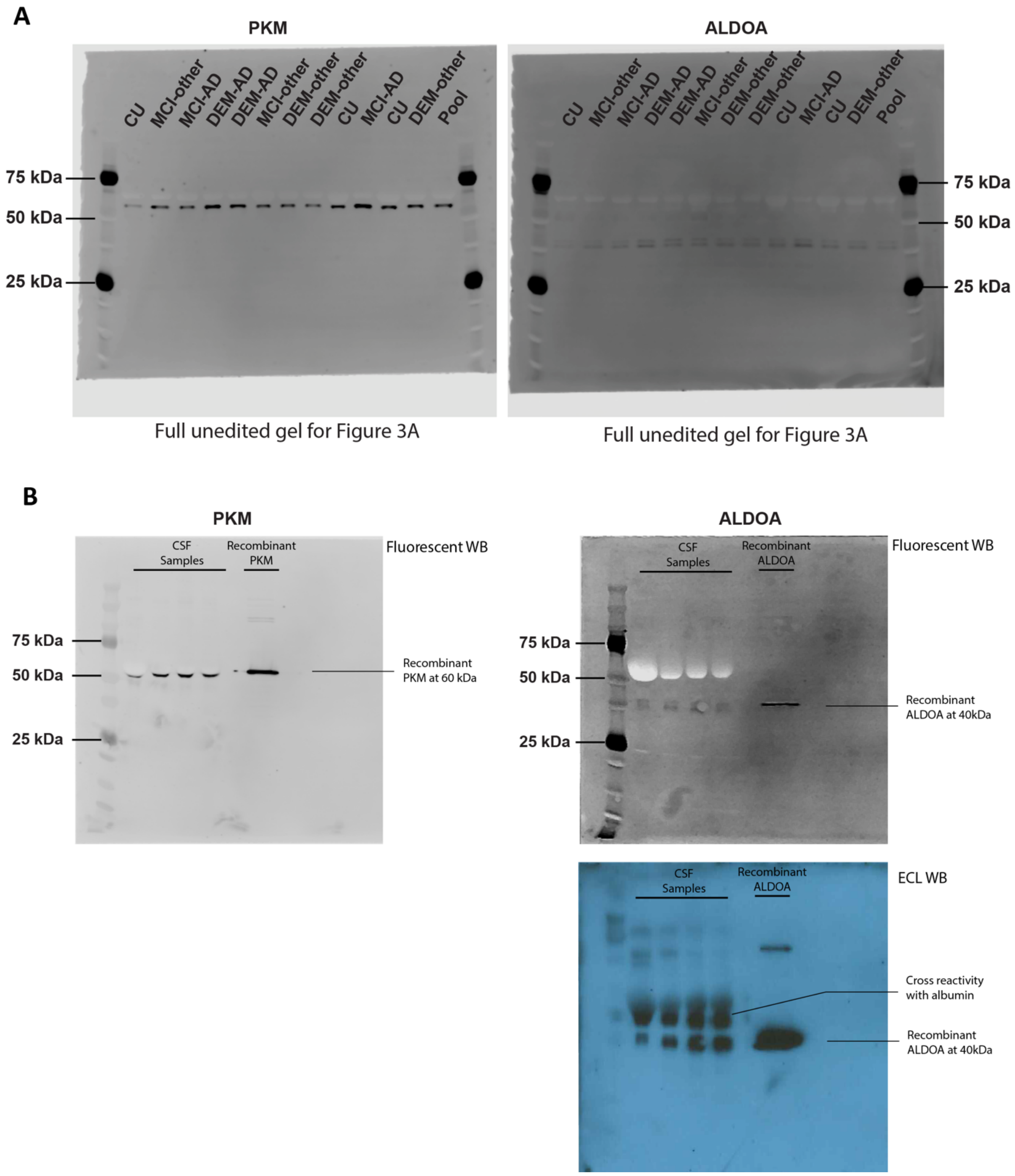

Supplementary Figure 4: A) *Examples of full blots for ALDOA and PKM. For PKM a single band was detected at the expected 60 kDa size. For ALDOA a double banded pattern was detected at the expected 40 kDa size. This suggests the potential presence of two variants of full-length ALDOA in CSF. Total protein content that was loaded onto the gel was normalized across all samples and each blot contained a pooled sample to normalize band intensities to.* ***B)*** *Full-length size of ALDOA and PKM was confirmed using recombinant protein. CSF samples were loaded on a gel with either recombinant ALDOA or PKM. For PKM, the band observed in CSF samples showed at the same height as recombinant PKM, at the expected 60 kDa size. For ALDOA, the double banded pattern in CSF samples showed at the same height as recombinant ALDOA at the expected 40 kDa size. To intensify the bands for ALDOA the same ALDOA blot was also analysed with enhanced chemiluminescence (ECL). This verified the presence of ALDOA bands at the height of recombinant protein, but also resulted in cross-reactivity with albumin.*
